# CDK1 and CDC20 overexpression in patients with colorectal cancer are associated with poor prognosis: evidence from integrated bioinformatics analysis

**DOI:** 10.1186/s12957-020-01817-8

**Published:** 2020-03-04

**Authors:** Jianxin Li, Yinchun Wang, Xin Wang, Qingqiang Yang

**Affiliations:** Department of Gastrointestinal Surgery, The Affiliated Hospital of Southwest Medical University, Luzhou, 646000 Sichuan People’s Republic of China

**Keywords:** Colorectal cancer, Hub genes, Bioinformatics analysis, Cyclin-dependent kinase 1, Cell division cycle 20 homolog

## Abstract

**Background:**

Colorectal cancer (CRC) is one of the most common malignancies of the digestive system, which causes severe financial burden worldwide. However, the specific mechanisms involved in CRC are still unclear.

**Methods:**

To identify the significant genes and pathways involved in the initiation and progression of CRC, the microarray dataset GSE126092 was downloaded from Gene Expression Omnibus (GEO) database, and then, the data was analyzed to identify differentially expressed genes (DEGs). Subsequently, the Gene Ontology (GO) annotation and Kyoto Encyclopedia of Genes and Genomes (KEGG) pathway analysis were performed on these DEGs using the DAVID database, and the protein-protein interaction (PPI) network was constructed using the STRING database and analyzed using the Cytoscape software. Finally, hub genes were screened, and the survival analysis was performed on these hub genes using the Kaplan-Meier curves in the cBioPortal database.

**Results:**

In total, 937 DEGs were obtained, including 316 upregulated genes and 621 downregulated genes. GO analysis revealed that the DEGs were mostly enriched in terms of nuclear division, organelle fission, cell division, and cell cycle process. KEGG pathway analysis showed that the DEGs were mostly enriched in cell cycle, oocyte meiosis, cytokine-cytokine receptor interaction, and cGMP-PKG signaling pathway. The PPI network comprised 608 nodes and 3100 edges, and 4 significant modules and 10 hub genes with the highest degree were identified using the Cytoscape software. Finally, survival analysis showed that overexpression of CDK1 and CDC20 in patients with CRC were statistically associated with worse overall survival.

**Conclusions:**

This bioinformatics analysis revealed that CDK1 and CDC20 might be candidate targets for diagnosis and treatment of CRC, which provided valuable clues for CRC.

## Introduction

Colorectal cancer (CRC) is one of the leading causes of malignancies, which causes severe financial burden worldwide [1, 2]. The morbidity of CRC is elevating during the past years, and its age of onset has gradually decreased [3]. The prognosis of patients with CRC is primarily associated with the staging of the primary tumor, patients with CRC at early stages may be cured, and the 5-year relative survival rate was 90%. However, the prognosis of CRC cases at advanced stages is poor, and recurrence and metastasis are the principal causes of death, despite improvements in surgery-based integrated treatment. Unfortunately, the majority of CRC patients are diagnosed at stages when the cancer cells have metastasized to other organs in the body [4, 5]. Although numerous efforts have been taken to understand the molecular mechanisms involved in the initiation and progression of CRC, the underlying molecular mechanisms and critical genes are still not completely cleared, and the morbidity and mortality of CRC are still rising year by year [6]. Therefore, there is still a need to explore more potential biomarkers for targeted therapy, early diagnosis, and prognosis evaluation of CRC.

The carcinogenesis and progression mechanisms of CRC involve interconnections between environmental and genetic factors; with the rapid development of gene sequencing technology and bioinformatics analysis technology, many researchers have begun to search for the significant differentially expressed genes of CRC through the data mining from online databases, such as the Gene Expression Omnibus (GEO) and The Cancer Genome Atlas (TCGA) [7]. Online databases can obtain expression information of numerous genes simultaneously, and these genes were analyzed to explore the significant genetic alterations associated with the initiation and progression of CRC [8]. Reanalyzing those datasets may find some meaningful information for new research, which provides efficient approaches to identify some potential early diagnostic biomarkers and therapeutic targets for the patients with CRC.

In the present study, the differentially expressed genes between CRC samples and adjacent noncancerous samples were obtained by mining gene expression microarray dataset GSE126092, and the Gene Ontology (GO) annotation and Kyoto Encyclopedia of Genes and Genomes (KEGG) pathway analysis of the DEGs were performed by using the Database for Annotation, Visualization and Integrated Discovery (DAVID) online tool. A protein-protein interaction (PPI) network of DEGs was constructed using the Search Tool for the Retrieval of Interacting Genes (STRING) database and analyzed using the Cytoscape software, and the hub genes were identified. In addition, the survival of patients with hub genes abnormal expression was analyzed using the cBioPortal database.

## Materials and methods

### Microarray data acquisition

Gene expression profiles of GSE126092 [9] were acquired from the National Center for Biotechnology Information Gene Expression Omnibus database (NCBI GEO, http://www.ncbi.nlm.nih.gov/geo) [10]; GSE126092 consisted of 10 paired CRC and adjacent noncancerous tissues, which was based on the GPL21047 platform (Agilent-074348 Human LncRNA v6 4X180K).

### Identification of DEGs

Before the analysis of DEGs between CRC and adjacent noncancerous tissues, the probe identification numbers of GSE126092 were transformed into gene symbols. When multiple probes corresponded to the same gene, the significant expression value was taken as the gene expression value. After that, the DEGs in the microarray were screened out using limma package in R V3.6.2 (http://www.bioconductor.org/) [11]; the cutoff conditions were set to adjusted *P* value < 0.05 and absolute value of log fold change (log_2_ FC) ≥ 2.

### Gene ontology and pathway enrichment analysis

To further clarify the potential functional annotation and pathway enrichment associated with the DEGs, Gene Ontology (GO) analysis including biological process, cellular component, and molecular function and Kyoto Encyclopedia of Genes and Genomes (KEGG) pathway analysis were completed with the Database for Annotation, Visualization and Integrated Discovery (DAVID, http://david.abcc.ncifcrf.gov/) (version 6.8) [12, 13]. DAVID is an online tool with gene annotation, visualization, gene ID conversion, and integrated discovery function, and thus can provide the biological significance of genes. Only terms with *P* values of <0.05 and the number of enriched genes ≥ 2 were considered statistically significant.

### Integration of protein-protein interaction network and module analysis

The protein-protein interaction (PPI) network of DEGs was constructed using the online database Search Tool for the Retrieval of Interacting Genes (STRING, http://string-db.org) (version 11.0) [14], and a confidence score of ≥ 0.4 was set as the threshold. The protein nodes which have no interaction with other proteins were removed. Furthermore, to screen the significant modules and hub genes from the PPI network, the PPI network was analyzed using the Cytoscape software (http://www.cytoscape.org/) (version 3.7.2) [15]. The MCODE plug-in was used to select significant clustering modules with the criteria of MCODE score > 3 and the number of nodes > 10, and GO and KEGG analyses of the genes in these modules were performed using DAVID. Subsequently, the CytoHubba plug-in was used to screen the PPI network, and the top 10 genes, which were the hub genes in the CRC, were identified using the degree algorithm.

### Survival analysis and validation of gene expression

In order to identify the potential prognostic role of these hub genes, the survival analysis of hub genes was performed using the Kaplan-Meier curves in the cBioPortal database (http://www.cbioportal.org) [16], and log rank test *P* value < 0.05 being the threshold of statistical significance. Then, the UCSC Cancer Genomics Browser (https://genome-cancer.ucsc.edu/) was used for hierarchical clustering of these hub genes [17]. Finally, a comparison of expression of these genes in multiple databases was analyzed using the online database Oncomine (http://www.oncomine.com) [18].

## Results

### Identification of DEGs in CRC

**Fig. 1:**
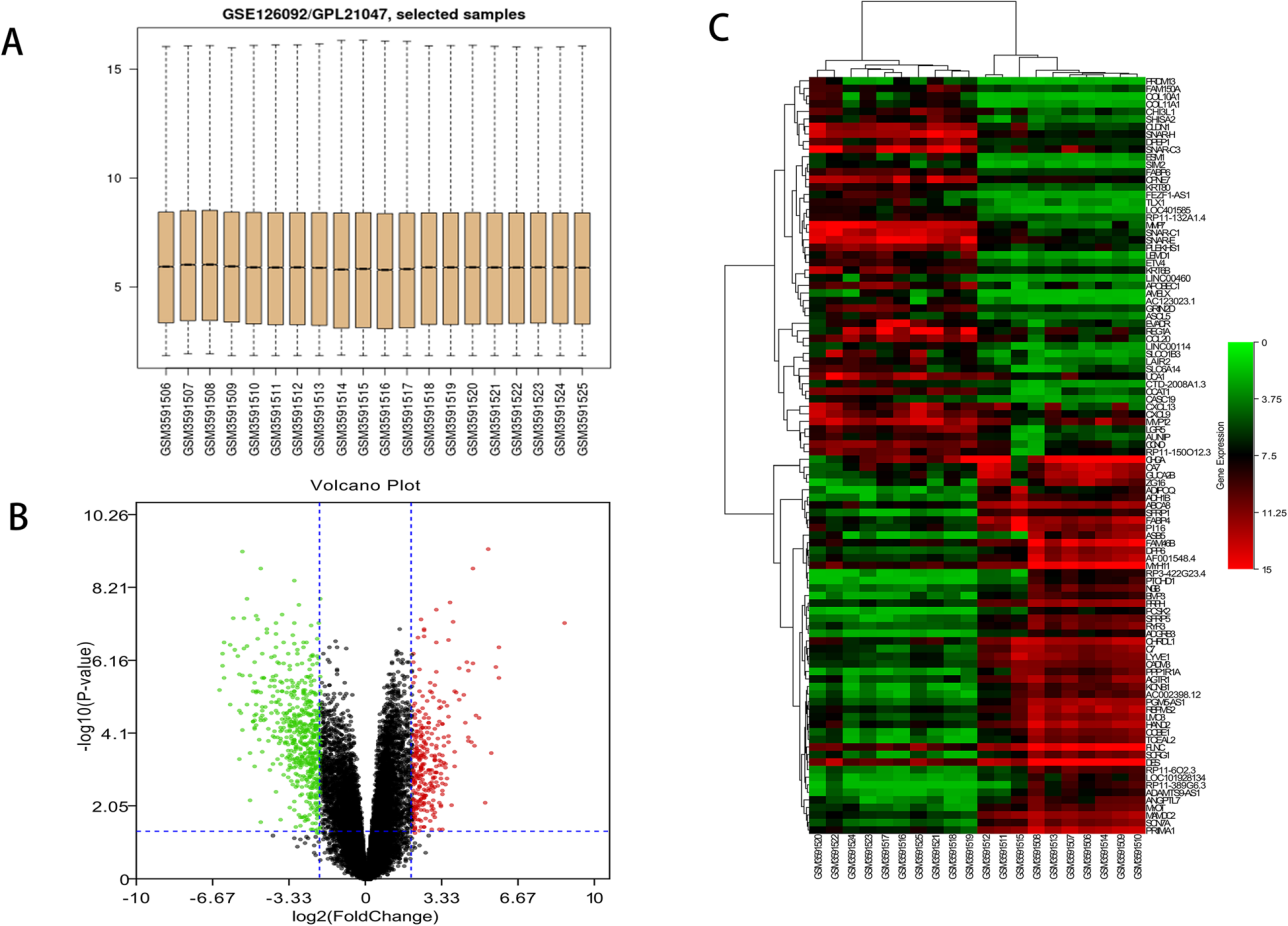
Identification of differentially expressed genes. **a** Boxplot of the distribution of each sample in GSE126092. **b** Volcano plot of DEGs. The red dots represent the upregulated genes, the green dots represent the downregulated genes, and the black dots represent genes with no significant difference in expression. **c** Heatmap of the top 100 DEGs. Red represents upregulated genes, and green represents downregulated genes

In the present study, the row data of GSE126092 dataset, including 10 paired CRC and adjacent noncancerous tissues, was downloaded from the GEO database. The median value of each sample was normalized (Fig. 1a). A total of 937 genes were identified, including 316 upregulated genes and 621 downregulated genes. The volcano plot of DEGs was presented in Fig. 1b, and the expression heat map of the top 50 upregulated and the top 50 downregulated DEGs were presented in Fig. 1c.

### GO and KEGG enrichment analyses of DEGs

To further explore the biological function of the DEGs, the DAVID database was used to perform GO and KEGG enrichment analysis. As shown in Table 1, in the biological process (BP) group, upregulated DEGs were primarily enriched in nuclear division, organelle fission, cell division, and cell cycle process, while downregulated DEGs were primarily enriched in muscle system process, regulation of system process, and system process. In the cellular component (CC) group, upregulated DEGs were primarily enriched in chromosome and chromosome centromeric region, while downregulated DEGs were primarily enriched in neuron projection and neuron part. In the molecular function (MF) group, upregulated genes were primarily enriched in microtubule motor activity, chemokine activity, and motor activity, while downregulated DEGs were primarily enriched in ion channel activity, channel activity, and passive transmembrane transporter activity. Moreover, KEGG pathway analysis results showed that the upregulated DEGs were significantly enriched in cell cycle, oocyte meiosis, and cytokine-cytokine receptor interaction, while downregulated DEGs were significantly enriched in cGMP-PKG signaling pathway, hypertrophic cardiomyopathy, and adipocytokine signaling pathway.

**Table 1:** Gene Ontology and KEGG pathway analysis of DEGs in colorectal cancer

Expression	Category	Term	Count	*P* value
Upregulated	GOTERM_BP_FAT	GO:0000280~nuclear division	40	1.34E−18
	GOTERM_BP_FAT	GO:0048285~organelle fission	40	1.23E−17
	GOTERM_BP_FAT	GO:0051301~cell division	37	2.68E−16
	GOTERM_BP_FAT	GO:0022402~cell cycle process	56	4.22E−16
	GOTERM_BP_FAT	GO:0007049~cell cycle	61	3.43E−15
	GOTERM_CC_FAT	GO:0005694~chromosome	41	1.95E−11
	GOTERM_CC_FAT	GO:0000775~chromosome, centromeric region	18	4.41E−10
	GOTERM_CC_FAT	GO:0005819~spindle	22	4.71E−10
	GOTERM_CC_FAT	GO:0044427~chromosomal part	36	5.61E−10
	GOTERM_CC_FAT	GO:0098687~chromosomal region	22	5.14E−09
	GOTERM_MF_FAT	GO:0003777~microtubule motor activity	10	4.37E−07
	GOTERM_MF_FAT	GO:0008009~chemokine activity	7	2.38E−05
	GOTERM_MF_FAT	GO:0003774~motor activity	10	4.01E−05
	GOTERM_MF_FAT	GO:0045236~CXCR chemokine receptor binding	5	4.08E−05
	GOTERM_MF_FAT	GO:0005125~cytokine activity	12	7.12E−05
	KEGG_PATHWAY	hsa04110:Cell cycle	15	9.41E−10
	KEGG_PATHWAY	hsa04114:Oocyte meiosis	10	1.81E−05
	KEGG_PATHWAY	hsa04914:Progesterone-mediated oocyte maturation	9	2.17E−05
	KEGG_PATHWAY	hsa04060:Cytokine-cytokine receptor interaction	13	1.05E−04
	KEGG_PATHWAY	hsa05322:Systemic lupus erythematosus	8	0.002278
Downregulated	GOTERM_BP_FAT	GO:0003012~muscle system process	43	1.93E−15
	GOTERM_BP_FAT	GO:0044057~regulation of system process	45	1.99E−13
	GOTERM_BP_FAT	GO:0006936~muscle contraction	33	3.28E−11
	GOTERM_BP_FAT	GO:0003008~system process	96	1.06E−10
	GOTERM_BP_FAT	GO:0007399~nervous system development	100	2.43E−10
	GOTERM_CC_FAT	GO:0043005~neuron projection	60	1.19E−10
	GOTERM_CC_FAT	GO:0042383~sarcolemma	19	5.73E−10
	GOTERM_CC_FAT	GO:0097458~neuron part	67	4.47E−08
	GOTERM_CC_FAT	GO:0044449~contractile fiber part	22	4.57E−08
	GOTERM_CC_FAT	GO:0030425~dendrite	34	7.24E−08
	GOTERM_MF_FAT	GO:0005216~ion channel activity	32	5.05E−08
	GOTERM_MF_FAT	GO:0015267~channel activity	34	5.49E−08
	GOTERM_MF_FAT	GO:0022803~passive transmembrane transporter activity	34	5.77E−08
	GOTERM_MF_FAT	GO:0022838~substrate-specific channel activity	32	1.16E−07
	GOTERM_MF_FAT	GO:0005539~glycosaminoglycan binding	20	6.73E−07
	KEGG_PATHWAY	hsa04022:cGMP-PKG signaling pathway	13	8.87E−04
	KEGG_PATHWAY	hsa05410:Hypertrophic cardiomyopathy (HCM)	9	9.58E−04
	KEGG_PATHWAY	hsa05414:Dilated cardiomyopathy	9	0.001557
	KEGG_PATHWAY	hsa04920:Adipocytokine signaling pathway	8	0.002302
	KEGG_PATHWAY	hsa04261:Adrenergic signaling in cardiomyocytes	11	0.00334

The top five terms of GO and KEGG analysis were selected according to *P* value. *GO* Gene Ontology, *BP* biological process, *CC* cellular component, *MF* molecular function, *KEGG* Kyoto Encyclopedia of Genes and Genomes

### Integration of protein-protein interaction network and module analysis

**Fig. 2:**
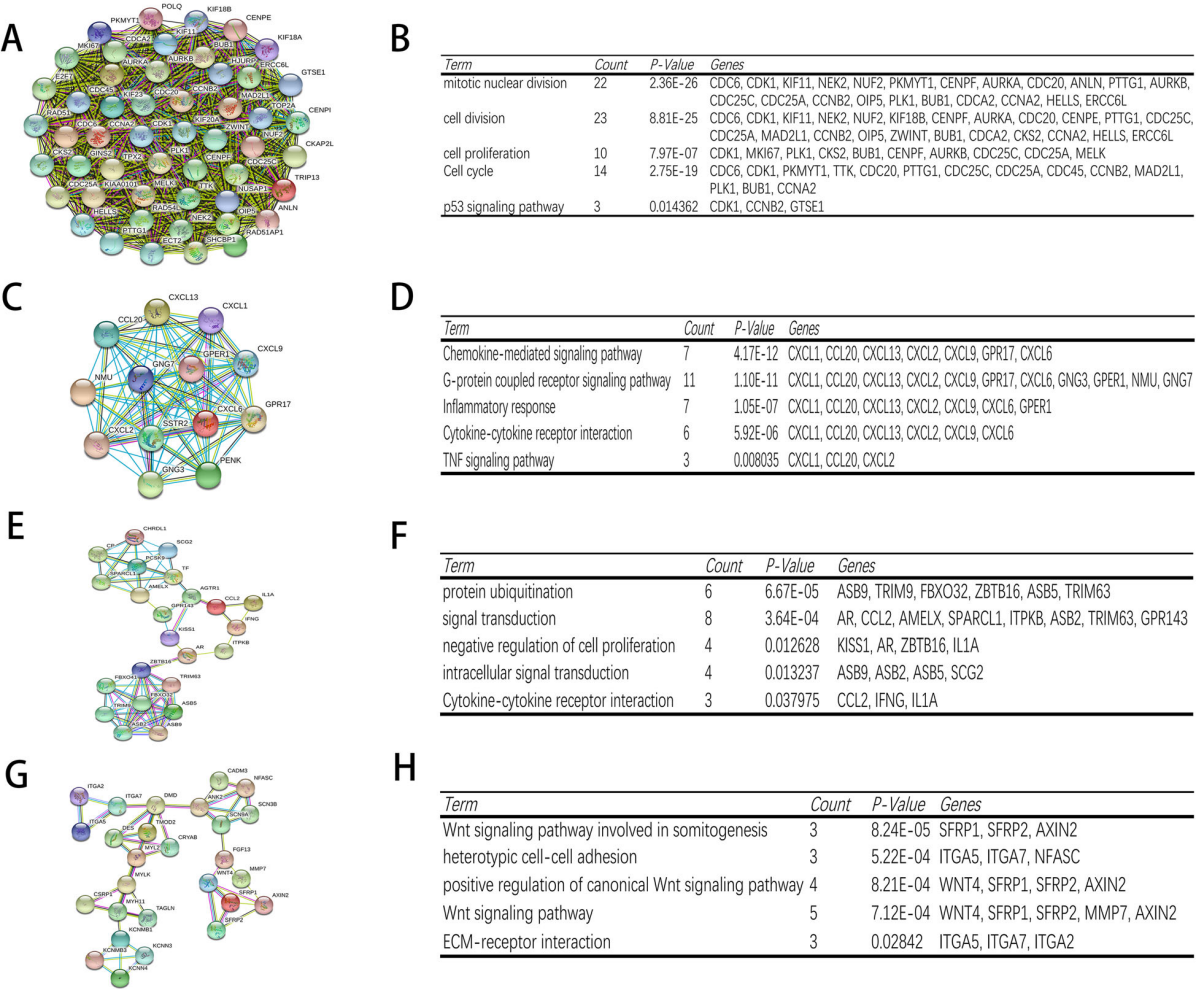
The top four modules from the PPI network and the functional annotation of the DEGs involved in the modules. **a** Module 1. **b** The functional annotation of module 1. **c** Module 2. **d** The functional annotation of module 2. **e** Module 3. **f** The functional annotation of module 3. **g** Module 4. **h** The functional annotation of module 4

The STRING online database was used to construct a PPI network consisting of 608 nodes and 3100 edges, and this network was then analyzed using the Cytoscape software. Four significant clustering modules were selected using the MCODE plug-in, and the functional annotation of the DEGs involved in these modules was analyzed (Fig. 2). Clustering module 1 consisting of 50 nodes and 1129 edges, the genes in module 1 were primarily associated with mitotic nuclear division, cell division, and P53 signaling pathway. Clustering module 2 consisting of 13 nodes and 78 edges, the genes in module 2 were primarily associated with chemokine-mediated signaling pathway, G protein-coupled receptor pathway, and TNF signaling pathway. Clustering module 3 consisting of 23 nodes and 62 edges, the genes in module 3 were primarily associated with protein ubiquitination, signal transduction, and cytokine-cytokine receptor interaction. Clustering module 4 consisting of 27 nodes and 48 edges, the genes in module 4 were primarily associated with Wnt signaling pathway, heterotypic cell-cell adhesion, and ECM-receptor interaction. Furthermore, the top 10 hub genes (CDK1, CDC20, AURKA, PLK1, AURKB, CDC6, KIF11, CCNA2, CENPE, and MKI67) were screened using the degree algorithm of the CytoHubba plug-in; the full name and function of these hub genes are listed in Table 2. Among these hub genes, CDK1 and CDC20 showed the highest degree.

**Table 2:** Functional roles of 10 hub genes

No.	Gene symbol	Full name	Function
1	CDK1	Cyclin-dependent kinase 1	Plays a key role in the control of the eukaryotic cell cycle by modulating the centrosome cycle as well as mitotic onset
2	CDC20	Cell division cycle protein 20 homolog	Required for full ubiquitin ligase activity of the anaphase promoting complex/cyclosome and may confer substrate specificity upon the complex
3	AURKA	Aurora kinase A	Mitotic serine/threonine kinase that contributes to the regulation of cell cycle progression.
4	PLK1	Polo-like kinase 1	Serine/threonine-protein kinase that performs several important functions throughout M phase of the cell cycle
5	AURKB	Aurora kinase B	Serine/threonine-protein kinase component of the chromosomal passenger complex (CPC), a complex that acts as a key regulator of mitosis
6	CDC6	Cell division control protein 6 homolog	Involved in the initiation of DNA replication. Also participates in checkpoint controls that ensure DNA replication is completed before mitosis is initiated
7	KIF11	Kinesin family member 11	Motor protein required for establishing a bipolar spindle during mitosis. Required in nonmitotic cells for transport of secretory proteins from the Golgi complex to the cell surface
8	CCNA2	Cyclin A2	Cyclin which controls both the G1/S and the G2/M transition phases of the cell cycle
9	CENPE	Centromere-associated protein E	Microtubule plus-end-directed kinetochore motor which plays an important role in chromosome congression, microtubule-kinetochore conjugation, and spindle assembly checkpoint activation
10	MKI67	Marker of proliferation Ki-67	Required to maintain individual mitotic chromosomes dispersed in the cytoplasm following nuclear envelope disassembly

### Survival analysis and validation of gene expression

**Fig. 3:**
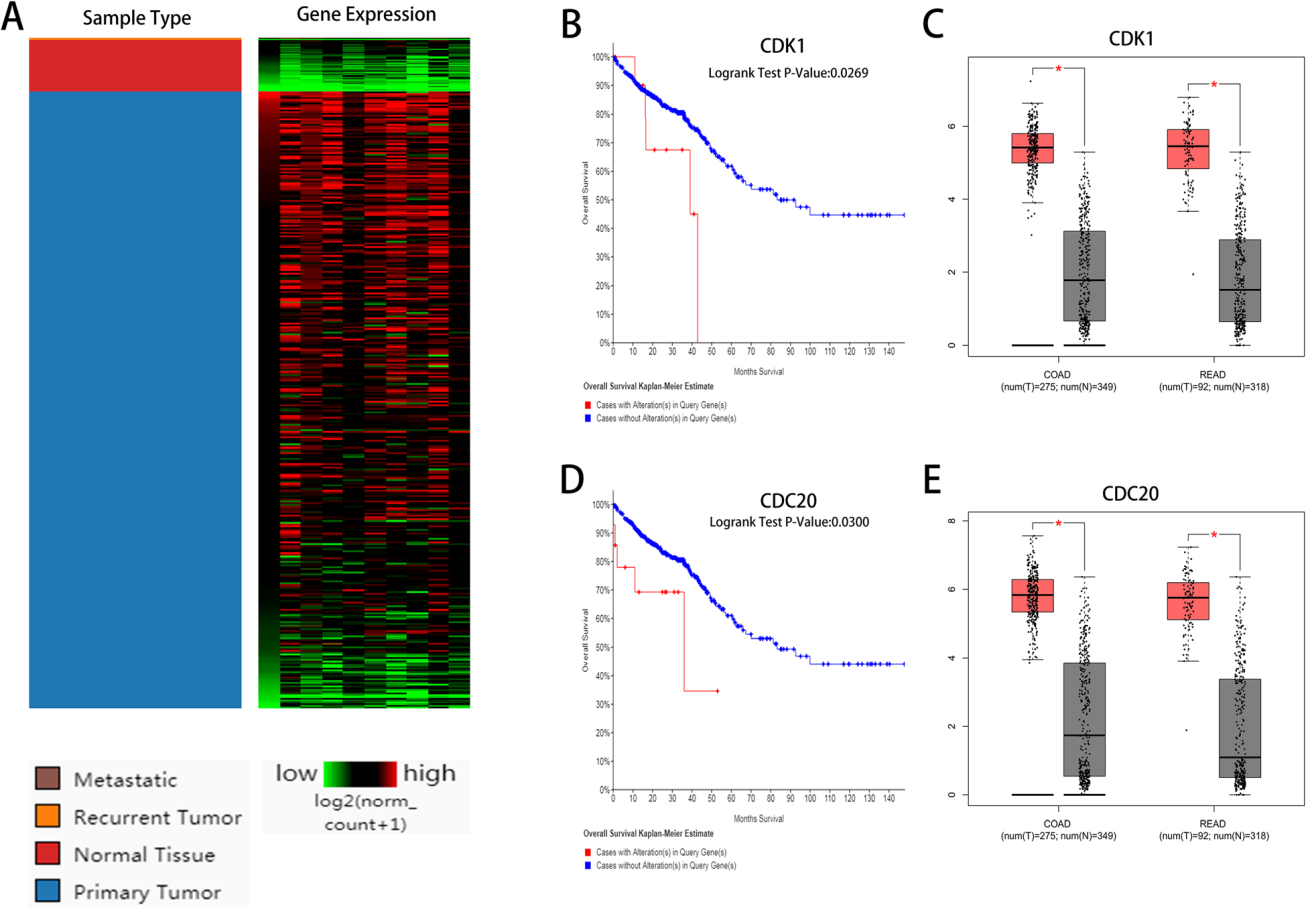
Hierarchical clustering and survival analysis of hub genes. **a** Hierarchical clustering of hub genes. Survival analysis of **b** CDK1 and **d** CDC20 genes. Expression levels of **c** CDK1 and **e** CDC20 genes in the tumor and normal groups. Red represents tumor groups, gray represents normal groups, *x*-axis represents the groups, *y*-axis represents the expression level of the gene, and asterisk (*) represents *P* < 0.05

**Fig. 4:**
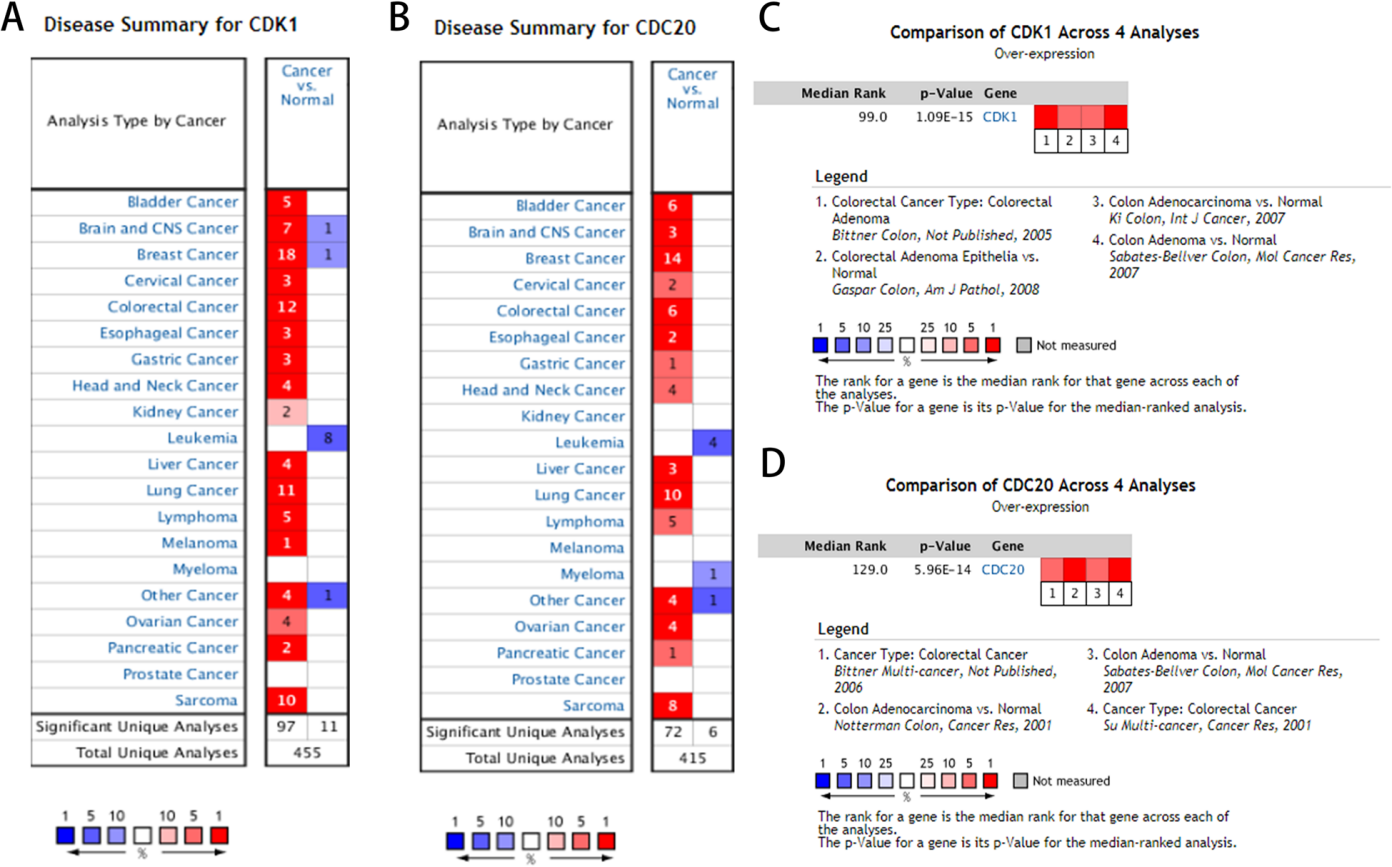
Oncomine analysis of CDK1 and CDC20 gene expression in cancers including CRC. The expression of **a** CDK1 and **b** CDC20 in various tumor tissue types. Heat maps of **c** CDK1 and **d** CDC20 gene expression in multiple CRC tissues vs. normal tissues

To investigate the expression of hub genes in CRC, a hierarchical clustering analysis was performed using the UCSC Cancer Genomics Browser, revealing that these 10 hub genes were highly expressed in most CRC samples (Fig. 3a). Subsequently, the survival analysis of these 10 hub genes was performed using the Kaplan-Meier curves in the cBioPortal database. The results revealed that patients with CDK1 and CDC20 alteration showed worse overall survival (Fig. 3b, d), and the expression levels of CDK1 and CDC20 genes were differentially expressed in CRC samples and noncancerous samples (Fig. 3c, e). However, the differential expression of AURKA, PLK1, AURKB, CDC6, KIF11, CCNA2, CENPE, and MKI67 were not significantly associated with the survival of CRC patients (*P* > 0.05). Oncomine analysis revealed that CDK1 and CDC20 expression were obviously elevated in most cancers, especially in breast cancer, colorectal cancer, and lung cancer (Fig. 4a, b). In addition, CDK1 and CDC20 were significantly overexpressed in CRC in multiple CRC datasets (Fig. 4c, d). These findings indicated that CDK1 and CDC20 might be candidate targets for diagnosis and treatment of CRC.

## Discussion

Colorectal cancer (CRC) is the third most common malignant tumor in the world, and the morbidity of CRC has gradually increased over the past decades [1]. The initiation and progression of CRC are involved; although numerous studies have been conducted to reveal the molecular mechanisms of CRC initiation and progression over the past decade, the morbidity of CRC is still very high. Therefore, there is a further need to explore the molecular mechanisms of CRC for early diagnosis and treatment of the disease. The development of microarray technology and gene sequencing technology has helped to understand the pathogenesis of colorectal cancer at the molecular level.

Therefore, the present study aims to investigate the causes of CRC initiation and progression, and gene expression data of 10 paired CRC and adjacent noncancerous tissues were obtained from the GEO database. A total of 937 DEGs, including 316 upregulated genes and 621 downregulated genes, were identified by bioinformatics analysis. In order to further explore the biological function of these DEGs, functional annotation analysis of these DEGs was performed by using the DAVID database. GO analysis revealed that the upregulated DEGs were mostly associated with nuclear division, organelle fission, cell division, and cell cycle process, while downregulated DEGs were primarily associated with the muscle system process, regulation of system process, and system process. KEGG pathway analysis revealed that the upregulated DEGs were mostly associated with cell cycle and cytokine-cytokine receptor interaction, while downregulated DEGs were significantly enriched in the cGMP-PKG signaling pathway. These results above showed that the upregulated DEGs might influence CRC initiation and progression by regulating cell division and cell cycle process, and the downregulated DEGs may be linked to the initiation and progression of CRC by regulating signal transduction pathways. Previous studies indicated that dysregulation of the mechanisms that control cell proliferation, differentiation, and apoptosis has long been considered as a mechanism for cancer cells to gain a growth advantage over noncancerous cells [19]. Cytokines are involved in regulating immune response, stimulating cell activation, proliferation, and differentiation, and many studies have pointed out that the dysregulation of cytokine, such as TNFα, IL-1β, and IL-22, was associated with the pathological process of CRC [20, 21]. Recent researches also pointed out that the cGMP/PKG cascade is recognized as an endogenous apoptotic pathway in numerous cancer types, including CRC [22]. Browning et al. [23] indicated that PKG1 could block tumor growth and angiogenesis in xenografts by reducing the production of VEGF by tumor cells, and cGMP-dependent protein kinase may be potential targets for colon cancer prevention and treatment.

We constructed a PPI network with all DEGs, and the module analysis of the PPI networks suggested that the carcinogenesis and progression of CRC were associated with cell cycle, P53 signaling pathway, chemokine-mediated signaling pathway, TNF signaling pathway, protein ubiquitination, Wnt signaling pathway, and ECM-receptor interaction. P53 functions as one of the most critical tumor suppressor gene, which is the most common mutational gene in human cancer [24]. Cooks et al. [25] reported that the dysregulation of the P53 signaling pathway might exacerbate the inflammatory response and protects the mutant cells from the clearance of the immune system by enhancing the activity of NF-kB, which promoted the carcinogenesis and progression of chronic inflammation and inflammation-associated CRC. Schulz-Heddergott et al. [26] revealed that the decrease of p53 mutation level could inhibit the carcinogenesis and progression of CRC. Mitchell et al. [27] indicated that the abnormal expression of chemokines or chemokine receptors in CRC might contribute to identify the molecular characteristics of tumors and predict the clinical prognosis of patients; several chemokines and chemokine receptors have been shown to promote CRC metastasis. Aberrations in the ubiquitin system also have been reported to be associated with various diseases, including cancer, for example, with elevated expression of Cathepsin D and Ubiquitin C-terminal hydrolase-L1 in tumor cells, the incidence of lymph node metastasis in CRC also has increased [28]. Previous studies have reported that the Wnt signaling pathway was altered in 93% of all tumors, approximately 90% of CRC patients with the dysregulation of the Wnt signaling pathway [29]. Moreover, de Sousa e Melo and Vermeulen [30] pointed out that the aberrant regulation of Wnt signaling is associated with chemotherapeutic resistance in CRC patients. ECM-receptor interaction pathway also plays a critical role in the process of proliferation, differentiation, and metastasis of cancer cells; Rahbari et al. [31] reported that the ECM could promote the metastasis of CRC by inducing epithelial-mesenchymal transition (EMT) in tumor cells.

After further analysis of the PPI network, 10 hub genes (CDK1, CDC20, AURKA, PLK1, AURKB, CDC6, KIF11, CCNA2, CENPE, and MKI67) with the highest degrees of interaction were screened, and the expression levels of all these genes showed constant upregulation compared to the gene expression levels of the normal mucosa. Furthermore, results of survival analysis showed that the patients with high gene expression levels of CDK1 and CDC20 showed a worse prognosis. These results suggested that CDK1 and CDC20 are involved in the recurrence and metastasis of CRC, and these two genes may be novel targets for early diagnosis and therapy of CRC.

As a member of the cyclin-dependent kinases (CDKs) family, cyclin-dependent kinase 1 (CDK1) acts as a vital driver of cell cycle transition [32]. Over the past decade, a large number of researches have shown that dysregulation of CDK1 not only causes rapid tumor growth, but also leads to the spontaneous proliferation of cancer cells [33]. Previous studies have shown that CDK1 is involved in the progression of multiple types of cancer, including colorectal cancer, liver cancer, and lung cancer. Furthermore, the upregulation of CDK1 is associated with reduced survival time for these diseases [34–36]. Xue et al. [37] reported that the activity of CDK1 is highly elevated in CRC tissues compared to noncancerous tissues, and it predicts distant metastasis risk in CRC; CDK1 can promote the progression of CRC through phosphorylation of JAK1 to activate the JAK/STAT3 signaling pathway. Bury et al. [38] indicated that high gene expression levels of CDK1 stimulated the proliferation and migration of colorectal cancer cells, and inhibition of CDK1 activity by using inhibitors can inhibit the proliferation of colorectal cancer cells in vitro and in vivo. In addition, Zhang et al. [39] indicated that frequent overexpression of CDK1 in human CRCs is associated with the therapeutic target, and the therapeutic resistance of BRAF mutant human CRC can be suppressed by targeting CDK1.

Cell division cycle 20 homolog (CDC20), a homolog of the cell division cycle 20 protein in saccharomyces cerevisiae, has long been recognized as one of the significant regulatory components of the cell cycle and plays a significant role in carcinogenesis and progression of various malignancies [40]. Kidokoro et al. [41] reported that CDC20 is highly expressed in the vast majority of malignancies, including CRC, and the expression level of CDC20 is negatively regulated by p53, with the silencing of CDC20 significantly inhibiting cell growth in vitro. In addition, elevated CDC20 levels have been found to be associated with clinical stage, pathologic differentiation, and TNM stage in CRC, and patients with overexpression of CDC20 had a shorter overall survival than those with low expression of CDC20 [42]. Moreover, Hadjihannas et al. [43] reported that Wnt/β-catenin signaling pathway, which acts as a critical role in embryonic development, stem cell maintenance, and carcinogenesis, is regulated by CDC20 via controlling the levels of conduction protein during the cell cycle, and CDC20 knockdown inhibited the proliferation of CRC cells. These results suggested that high expression of CDC20 was an independent prognostic factor, and CDC20 serves as a potential prognostic biomarker for patients with CRC.

Consistent with our studies, previous studies also indicated that the abnormal expression of other hub genes in our study were associated with the initiation and progression of CRC. Aurora kinases A (AURKA) and Aurora kinases B (AURKB) were members of the Aurora kinase family, which serves as critical regulators of mammalian mitosis, and have been described to be associated with chromosomal instability, aggressive growth, and worse prognosis in various malignancies including CRC [44]. Goos et al. [45] revealed that AURKA overexpression is involved in the process of liver metastasis after colorectal cancer resection, and AURKA inhibitors could be developed as therapeutic agents for CRC. In addition, studies also have been reported that high gene expression levels of AURKB were significantly associated with decreased overall survival in patients with CRC [44]. Polo-like kinase 1 (PLK1) was one of the most significant member of the Polo-like kinase family and plays an important role in cell division and checkpoint regulation during mitosis; with the depleting of PLK1, the proliferation of WT p53-expressing CRC cells was inhibited [46]. Cell division control protein 6 homolog (CDC6), a central regulator of DNA replication and cell proliferation, has been reported to act as a potential oncogenic target in multiple tumors. CDC6 knockdown inhibited CRC cell malignant behaviors (e.g., growth, DNA synthesis, EMT) and oxaliplatin resistance in vitro [47]. Kinesin family member 11 (KIF11) belongs to the kinesin-like protein family, which encodes the motor protein. Imai et al. [48] reported that overexpression of KIF11 is an early event in the pathogenesis of CRC. However, the effect of KIF11 on the prognosis of patients with CRC remains unclear. There is also increasing evidence that cyclin A2 (CCNA2) could be a novel biomarker for diagnosis and therapy for CRC. Gan et al. [49] reported that CCNA2 was overexpressed in CRC tissues and cell lines, and CCNA2 knockdown significantly inhibited the proliferation of CRC cells by inhibiting cell cycle progression and inducing apoptosis. Centrosome-associated protein E (CENPE) is a kinesin-like motor protein, which accumulates in the G2 phase of the cell cycle [50]. Previous studies have shown that CENPE was dysregulated in various malignancies, such as epithelial ovarian cancer, prostate cancer, and breast cancer, and overexpression of CENPE was associated with promoting cell cycle progression and tumor cell growth [51]. Nevertheless, the molecular mechanism and prognostic value of CENPE in CRC remain unclear. Marker of proliferation Ki-67 (MKI67) is a proliferation-related nucleus protein gene that is widely expressed in proliferating cells [52]. Previous studies have reported the abnormally high expression of MKI67 in multiple types of cancer, including CRC, and found that MKI67 could be an independent prognostic biomarker in these types of cancer [53]. However, these genes above in our study were not significantly associated with the prognosis in CRC patients. Therefore, further investigation is required to elucidate the mechanism of these genes in the carcinogenesis, progression, and treatment of CRC.

## Conclusion

In conclusion, through integrated bioinformatics analysis, we identified hub genes and the association pathways involved in the initiation and progression of CRC. These findings could improve our understanding of the molecular mechanisms underlying the progression of CRC. Furthermore, the survival analysis of hub genes showed that overexpression of CDK1 and CDC20 were associated with poor survival in patients with CRC, suggesting that CDK1 and CDC20 may have potential as biomarker for CRC diagnosis, treatment, or prognosis determination. However, valuable clues provided by this study required further studies to elucidate its biological function in CRC.

## Data Availability

The datasets generated and/or analyzed during the current study are available in the Gene Expression Omnibus (GEO) repository (https://www.ncbi.nlm.nih.gov/geo/query/acc.cgi?acc=GSE126092).

## Abbreviations

AURKA: Aurora kinases A
AURKB: Aurora kinases B
BP: Biological process
CC: Cellular component
CCNA2: Cyclin A2
CDC20: Cell division cycle 20 homolog
CDC6: Cell division control protein 6 homolog
CDK1: Cyclin-dependent kinase 1
CENPE: Centrosome-associated protein E
CRC: Colorectal cancer
DEGs: Differentially expressed genes
GEO: Gene Expression Omnibus
GO: Gene Ontology
KEGG: Kyoto Encyclopedia of Genes and Genomes
KIF11: Kinesin family member 11
MF: Molecular function
MKI67: Marker of proliferation Ki-67
NCBI: National Center for Biotechnology Information
PLK1: Polo-like kinase 1
PPI: Protein-protein interaction
TCGA: The Cancer Genome Atlas
